# Clostridium Perfringens Epsilon Toxin Binds to Membrane Lipids and Its Cytotoxic Action Depends on Sulfatide

**DOI:** 10.1371/journal.pone.0140321

**Published:** 2015-10-09

**Authors:** Carles Gil, Jonatan Dorca-Arévalo, Juan Blasi

**Affiliations:** 1 Departament de Bioquímica i Biologia Molecular and Institut de Neurociències, Universitat Autònoma de Barcelona, Bellaterra, Catalunya, Spain; 2 Laboratory of Cellular and Molecular Neuroscience, Department of Pathology and Experimental Therapeutics, School of Medicine, Universitat de Barcelona, L’Hospitalet de Llobregat, Barcelona, Spain; 3 IDIBELL-Bellvitge Biomedical Research Institute, L’Hospitalet de Llobregat, Barcelona, Spain; Institute Pasteur, FRANCE

## Abstract

Epsilon toxin (Etx) is one of the major lethal toxins produced by *Clostridium perfringens* types B and D, being the causal agent of fatal enterotoxemia in animals, mainly sheep and goats. Etx is synthesized as a non-active prototoxin form (proEtx) that becomes active upon proteolytic activation. Etx exhibits a cytotoxic effect through the formation of a pore in the plasma membrane of selected cell targets where Etx specifically binds due to the presence of specific receptors. However, the identity and nature of host receptors of Etx remain a matter of controversy. In the present study, the interactions between Etx and membrane lipids from the synaptosome-enriched fraction from rat brain (P2 fraction) and MDCK cell plasma membrane preparations were analyzed. Our findings show that both Etx and proEtx bind to lipids extracted from lipid rafts from the two different models as assessed by protein-lipid overlay assay. Lipid rafts are membrane microdomains enriched in cholesterol and sphingolipids. Binding of proEtx to sulfatide, phosphatidylserine, phosphatidylinositol (3)-phosphate and phosphatidylinositol (5)-phosphate was detected. Removal of the sulphate groups via sulfatase treatment led to a dramatic decrease in Etx-induced cytotoxicity, but not in proEtx-GFP binding to MDCK cells or a significant shift in oligomer formation, pointing to a role of sulfatide in pore formation in rafts but not in toxin binding to the target cell membrane. These results show for the first time the interaction between Etx and membrane lipids from host tissue and point to a major role for sulfatides in *C*. *perfringens* epsilon toxin pathophysiology.

## Introduction

*Clostridium perfringens* is a gram-positive and spore-forming anaerobe, comprising five toxinotypes (A, B, C, D, and E) classified on the basis of the combinatorial production of four toxins of proteinaceous nature [1]. All five types produce alpha toxin, which is a hemolytic, necrotizing and lethal phospholipase C, whereas type B produces beta and epsilon toxins, type C produces beta toxin, type D produces epsilon toxin (Etx), and type E produces iota toxin. The A and, to a lesser extent, C strains are residents in the human gut [2], with ruminant animals being the natural hosts of the B and D strains [3]. Etx is one of the most potent bacterial toxins known [4], causing fatal enterotoxemia in livestock and, hence, heavy economic losses. The Etx-encoding gene (*etx*) is carried by large plasmids, some of them sharing homology with the enterotoxin-encoding plasmids found in type A strains [5]. Over-proliferation of *etx*-carrying *C*. *perfringens* in intestines under certain circumstances produces large amounts of Etx, which diffuses through all organs and accumulates preferentially in the brain and kidneys [6]. The lethal effect of Etx, characterized by sudden death and, in some cases, acute neurological signs [7], has mainly been associated with general edema, also leading to a glutamate-mediated exocytotoxic effect and neuronal death [7–9]. Etx binds to components of synaptosomal fractions [10], myelinic structures [11, 12], glial cells [13], granule neurons and oligodendrocytes [14] and causes demyelination [12]. After injections into mice, the toxin shows the capacity to cross the blood–brain barrier (BBB), enter the brain parenchyma [13, 15] and act on neuronal cells [16]. In addition, Etx also affects the renal system, producing cytotoxicity of epithelial distal tubule cells [17–19]. In fact, the MDCK cell line of renal origin is the most sensitive cell line to Etx [20] among the cell lines tested to date, and hence it has been widely used for the study of the cytotoxic effect of Etx. Effects on humans appear to be extremely rare, with a few reported cases of Etx production [21, 22] and two case studies which provide evidence of Etx-producing *C*. *perfringens* strains in patients with gas gangrene [23, 24]. Moreover, Etx is also cytotoxic to cultured human cells, including the human renal adenocarcinoma cell line ACHN [25], the human renal leiomyoblastoma cell line G-402 [26], and primary cultures of human renal tubular epithelial cells (HRTEC) [27].

The toxin is produced as a non-toxic precursor molecule (epsilon protoxin, proEtx) that is activated by proteolytic cleavage of amino and carboxy terminal peptides [28]. proEtx presumably binds to the same surface cell receptors as the full active molecule and can prevent its binding and further toxicity [11, 13].

The molecular mechanism of Etx cytotoxicity is apparently well characterized, with three defined steps: i) binding to a specific receptor on the surface of host cells, ii) oligomerization and formation of a heptameric pre-pore complex, and iii) insertion into the plasma membrane, producing an active pore [29]. These three steps lead to ionic imbalance across the membrane and death in host cells [30]. Thus, Etx has been included in the β-pore-forming toxin family (β-PFT), which is consistent with studies on the three-dimensional structure of the toxin that show similarities with aerolysin, a pore-forming toxin from *Aeromonas hydrophila* [31], and the ability of Etx to form channels in artificial lipid bilayers [32].

The high specificity of Etx towards defined cellular targets (i.e. MDCK cell line) compared to its relatively low binding to artificial membranes [33, 34] and the sensitivity of Etx binding to glycanases [10, 35], supports the existence of a glycoprotein as the receptor for Etx [3, 10, 11, 36]. The *in vitro* binding of Etx to the hepatitis A virus cellular receptor 1 (HAVCR1), together with its ability to facilitate Etx cytotoxicity, support the role of HAVCR1 as a putative receptor for Etx [25, 37]. In addition, there is evidence that more than one receptor molecule can be involved in the recognition and binding of the toxin to different cell targets [38]. Supporting this view, other proteins have been revealed as important elements for the cytotoxic activity of Etx. In particular, caveolin-1 and -2 potentiate Etx-induced cytotoxicity by promoting toxin oligomerization [39] while Myelin and Lymphocyte protein (MAL) is required for ETX cytotoxicity [40].

Besides the existence of a putative protein receptor, a suitable lipid environment is critical for the binding of Etx to a cell surface [35]. In fact, Etx oligomerizes and forms channels in artificial lipid membranes [32] although with much less efficiency than in sensitive cell lines such as MDCK [33]. In addition, Etx binds to elements situated in cholesterol- and sphingolipid-enriched domains from rat synaptosomes and MDCK cells and forms heptameric channels within the membrane, causing an increase in potassium ion-permeability [41–43]. However, it is relevant that the removal of cholesterol from mpkCCD_c14_ cell membrane impairs Etx oligomerization and pore formation, but does not block cellular ATP release and cell necrosis, suggesting an Etx cytotoxic mechanism independent of pore formation [44]. Moreover, although the effects of cholesterol depletion by a cholesterol synthesis inhibitor, lovastatin, and methyl-β-cyclodextrin treatment on Etx binding and heptamerization have been examined [43], the effects of other lipid constituents on Etx binding and heptamerization remain uninvestigated.

In the present study, Etx binding to lipids from detergent-resistant domains from two biological models (P2 fraction from rat brain and MDCK cells) and to pure membrane lipid components was investigated. Lipid binding partners of Etx were identified, and the essential role of sulfatide in the cytotoxic effect of Etx on MDCK cells was defined, although this lipid plays no role in Etx binding to target cells. Therefore, a direct link between Etx and its action on sulfatide-enriched cells, such as oligodendrocytes or Schwann cells, is proposed.

## Materials and Methods

### Preparation of P2 fraction from rat brain

The crude synaptosomal fractions (P2) were prepared from 4- to 6-week-old Sprague-Dawley rat brains, as described previously [45], with slight modifications. Animal samples were obtained according to a procedure approved by the Ethics Committee in Animal and Human Experimentation of the Universitat Autònoma de Barcelona (ref. 513R) following the European Communities Council Directives86/609/CEE, 91/628/CEE and 92/65/CE. The whole brain, without meninges, was homogenized in 40 vol. (w/v) phosphate buffer (pH 7.4) supplemented with 0.32 M sucrose. Homogenization was performed with 12 strokes (900 rev/min) of a Potter homogenizer with a Teflon pestle (0.1 ± 0.15 mm clearance). The homogenate was centrifuged at 1,000 g for 5 min at 4°C. The supernatant was then centrifuged at 12,000 g for 20 min, both centrifugations in a JA-25.5 rotor. The obtained pellet was enriched in synaptosomes, and also contained considerable amounts of myelin and mitochondria. The crude synaptosomal pellet obtained from one brain was gently resuspended in 10 mL of sodium buffer containing 140 mM NaCl, 5 mM KCl, 5 mM NaHCO_3_, 1 mM MgCl_2_, 1.2 mM Na_2_HPO_4_, 20 mM Hepes/NaOH, and 10 mM glucose, and the pH was adjusted to 7.4.

### Cloning, expression and purification of recombinant Etx and proEtx

Etx, proEtx, proEtx-GST and proEtx-GFP were produced as previously described [13, 46]. Briefly, the expression of the recombinant proteins was induced overnight in the presence of 1 mM isopropyl beta-D-thiogalactopyranoside (IPTG) at room temperature (RT), in 250 mL of LB medium containing 50 μg/mL of ampicillin. Cells were pelleted and resuspended in ice cold phosphate buffer (PB) 20 mM pH 7.5 with NaCl 250 mM, sonicated and centrifuged at 12,000g for 20 min. The resultant supernatant was incubated with Glutathione Sepharose 4B beads (GE Healthcare Life Sciences) for 1 h at 4°C. Finally, the recombinant proteins were eluted by thrombin cleavage in 20 mM PB, pH 7.5 containing 250 mM NaCl and 2.5 mM CaCl_2_. When required, proEtx was fully activated by incubation with trypsin-coated agarose beads for 30 min at RT (Sigma-Aldrich), thus obtaining the active Etx form. Alternatively, proEtx was labeled with DyLight 488 (Thermo Scientific) following the manufacturer’s instructions, for confocal microscopy analysis on MDCK cells. Protein concentration was determined by the Bradford method [47], using bovine serum albumin (BSA) as the standard. The purity of the recombinant proteins was checked by SDS-PAGE and Coomassie Blue staining.

### Detergent-resistant membrane extraction

Detergent-resistant membranes (DRM) are an experimental model commonly used to study the so-called ‘membrane rafts’, which are cholesterol- and sphingolipid-rich dynamic membrane microdomains that incorporate specific proteins. DRM were extracted as described previously [45]. To obtain DRM, the medium was removed and the synaptosomal P2 fraction or MDCK cells were rinsed with cold PBS and homogenized at 4°C with sodium buffer containing 1% Triton X-100 by end-over-end mixing. Thereafter, the extracts were adjusted to 45% sucrose, and overlaid with 7 mL of 35% sucrose in sodium buffer and 2 mL of 5% sucrose in sodium buffer, inside an ultracentrifugation tube. DRM fractions were isolated by ultracentrifugation at 35,000 rpm, for 18 h at 4°C, using a SW41 rotor (Beckman Instruments Inc.). The gradient was harvested in 12 fractions of 1 mL each. The cholesterol levels in each fraction were enzymatically determined in a Cobas-Mira automatic analyzer using a commercial reactive mixture specific for total cholesterol quantification (BioSystems, Spain) containing 35 mM PIPES, pH 7.0, 0.5 mM sodium cholate, 28 mM phenol, 0.2 U/mL cholesterol esterase, 0.1 U/mL cholesterol oxidase, 0.8 U/mL peroxidase, and 0.5 mM 4-aminoantipirine. Protein concentration in the gradient was determined according to the Bradford method [47]. The correct extraction of DRM was assessed by means of the presence of protein markers determined by western blot, using Flotillin-1 and Thy-1 as lipid raft markers, whereas Transferrin receptor was used as a marker of soluble membranes.

### Western-blot analysis

Samples of each fraction from the sucrose gradient were analyzed by SDS-PAGE followed by western blot analysis. The separated proteins were transferred to a Protran nitrocellulose membrane (Schleicher and Schuell; Dassel, Germany) using a Mini TransBlot Cell 3 (Bio-Rad, USA) at 100 V for 1 h. The blotting buffer used contained 25 mM Tris, 200 mM glycine and 10% methanol (v/v). The membrane filters were blocked for 1 h with Tris-buffered saline, supplemented with 0.1% Tween 20 and 5% (w/v) defatted powdered milk. Then, the membranes were incubated overnight with the indicated antibody diluted in blocking buffer. The antibody against Transferrin receptor was from Zymed Laboratories Inc. (San Francisco, CA, USA), the antibody against Flotilin-1 was from Abcam (Cambridge, UK) the antibody against Thy-1 (clone OX-7) was a gift from G. Schiavo (Cancer Research UK, London), the polyclonal antibodies against Caveolin-1 was from BD Transduction Laboratories and the antibody against Myelin Basic Protein was from Sigma-Aldrich. Next, the membrane filters were incubated for 1 h with a secondary antibody conjugated with horseradish peroxidase diluted in blocking buffer. Several washes with Tris-buffered saline/0.1% Tween 20 were performed between each step. The western blots were developed with luminol-based assay and visualized using a GeneGnome HR chemiluminescence detection system coupled to a CCD camera (Syngene; Cambridge, UK).

Etx oligomerization was monitored in MDCK cells incubated with Etx, basically according Fennessey *et al*. [39]. Briefly, MDCK cell monolayers were grown in 24 well plates and incubated for 30 min at 37°C with Etx (10 or 30 nM). After washing with PBS, cells were lysated with SDS-PAGE sample buffer and analyzed by western blot [48].

### Thin Layer Chromatography (TLC)

Lipids from samples of the DRM fractions were extracted according to the Bligh and Dyer method [49]. During the extraction, acetic acid (0.057 M) was added to the water phase, in order to increase the recovery of acidic phospholipids [50]. Briefly, 960 μL of chloroform/methanol (1:2), 400 μL of chloroform and 200 μL of 0.1 M acetic acid were added, in that order, to 200 μL of sample. After shaking the mixture, the tubes were centrifuged (1,000 rpm, 5 min), and the organic phase was extracted and evaporated using Speed-Vac. When necessary, evaporated samples were resuspended in 25 μL of chloroform/methanol (4:1) and resolved on silica high-performance TLC plates using chloroform/methanol/water (68:28:4) when sphingolipid detection was required, or using chloroform/methanol/NH_4_OH/water (40:48:5:10) in the case of phosphoinositides (PIs). Standards (sulfatide, sphingomyelin or monophosphorylated PIs, all from Sigma-Aldrich) were resolved in parallel and staining of lipids was performed using primuline.

### Protein-lipid overlay (PLO)

Lipids from samples of DRM fractions were extracted as in the case of TLC. Then, lipids were spotted onto a Hybond-C membrane. The membrane was subsequently air-dried, blocked with 1% defatted milk in PBS for 1 h and overlaid with 2.5 μg/mL of recombinant toxin in blocking buffer overnight. After three washes with PBS supplemented with 0.1% Tween 20, bound toxin was detected using a polyclonal specific IgG raised in rabbit [17] and subsequent chemiluminescence, as in the case of western blots. To determine the presence of sulfatide in DRM gradients, a monoclonal antibody against sulfatide was used [51]. In some analyses, commercial membranes with spotted lipids were also used (Membrane lipid strips and PIP strips from Echelon Biosciences, USA) or, alternatively, commercial lipids were spotted onto Hybond C membranes. These lipids were: 1,2-Diacyl-sn-glycero-3-phospho-L-serine, sulfatide and a phosphoinositide mix (PIs) from bovine brain (all from Sigma). When indicated, signal intensity was determined using the GeneTools software (Syngene; Cambridge, UK).

### Confocal microscopy on MDCK cells

MDCK cells (ATCC, CCL-34) were grown on coverslips to confluence in DMEM-F12 medium supplemented with L-Glutamine and antibiotics. Cells were washed three times with PBS, and fixed with 4% paraformaldehyde (PFA) for 12 min at RT. After washing with PBS, cells were treated for 1 h at 37°C with up to 1 U/mL of sulfatase from *Aerobacter aerogenes* (Sigma-Aldrich) or 10 μg/ml of arylsulfatase A (ARSA, Life Technologies) when indicated, and subsequently blocked by adding PBS containing 20% normal goat serum (NGS) for 1 h at RT and incubated either with 300 nM of proEtx-GFP or DyLight 488 conjugated-proEtx in PBS containing 1% normal goat serum for 1 h at RT. After three washes with PBS, the samples were stained with TO-PRO-3 (1:1000 dilution, Molecular Probes, Invitrogen) for 7 min, washed again and mounted with an aqueous mounting medium (Fluoromount, SIGMA). Slides were examined in a spectral confocal microscope (Leica TCS-SL; CCiTUB, Biology Unit of Bellvitge Campus).

### Measurement of cell viability

The cytotoxic effect of Etx on MDCK cells was measured using the MTS (3-(4,5-dimethylthiazol-2-yl)-5-(3-carboxymethoxyphenyl)-2-(4-sulfophenyl)-2H-tetrazolium) -based assay (Promega, Madison, USA). MDCK cells were grown in 96-well plates to confluence in DMEM F12 medium supplemented with L-Glutamine and antibiotics. Cells were incubated with medium containing sulfatase from *Aerobacter aerogenes*, at 0.1 or 0.2 U of enzyme per well (1–2 U/mL). After 90 min, Etx was added at 2 or 5 nM final concentration. After 30 min of incubation at 37°C, 20 μl of MTS assay was added to each well, incubated for 1 h at 37°C and the absorbance measured with an ELISA plate reader at 490 nM. Controls were obtained by omitting Etx in each condition (100% of cell viability) or by adding 100 μL of medium containing 0.1% Triton X-100 (100% of cell lethality).

## Results

### Etx and proEtx bind to lipids from DRM extracted with Triton X-100

**Fig 1:**
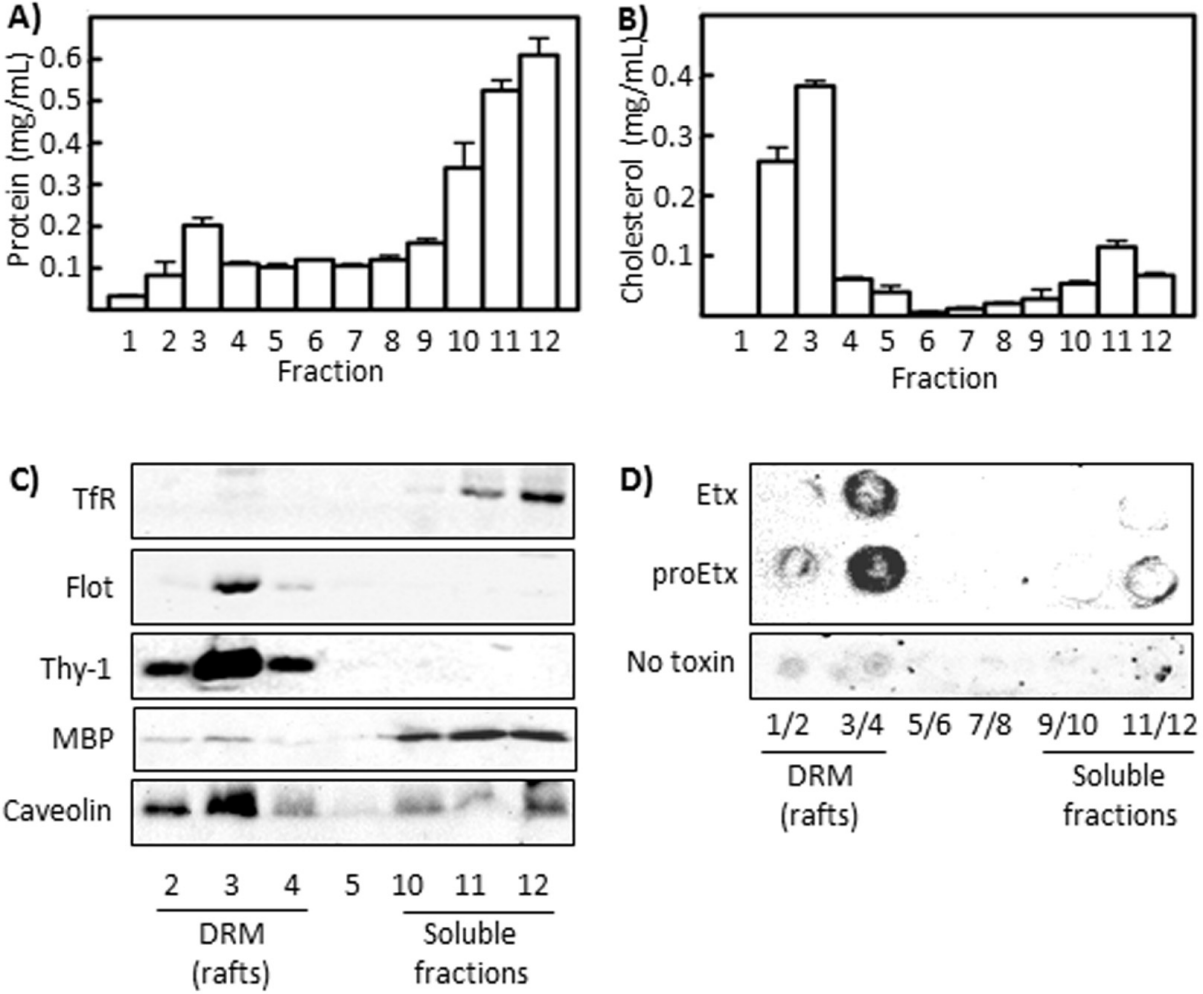
Etx and proEtx bind to lipids extracted from Triton X-100-DRM (*lipid rafts*) from rat brain. P2 fraction from brain cortex was treated with 1% Triton X-100 at 4°C, and detergent-resistant membranes (DRM) were isolated by ultracentrifugation in sucrose gradients. Twelve fractions were harvested after each gradient, and protein (A) and cholesterol (B) contents were determined, showing that the bulk of cholesterol is found in fractions two and three. Each histogram is the average ± SD from three independent experiments. (C) MBP, caveolin 1 and “lipid raft” marker proteins (Transferrin receptor, TfR, for soluble membranes; Flotillin-1 and Thy-1 for rafts) were detected by western blot. (D) Lipids, extracted under the Blight and Dyer method, were spotted onto a Hybond-C membrane and overlaid with 2.5 μg/mL of recombinant toxin (PLO assay). Bound toxin was detected with a specific polyclonal antibody and subsequent chemiluminescence. Results shown are representative of three independent experiments.

The specific binding and pore formation of Etx to DRM from synaptosomes and MDCK cells has been described previously, however the nature of the molecules necessary for binding has remained elusive [43]. Thus, in the present paper, an analysis of Etx and proEtx binding to lipids extracted from DRM isolated using Triton X-100 was conducted to better define and characterize the molecular nature and elements involved in Etx binding to biological targets. Since the synaptosome-enriched P2 fraction has already been used in the study of Etx binding to the nervous system [11], we extracted DRM from the P2 fraction from rat brain (Fig 1). The fractions corresponding to DRM showed relatively low levels of protein (Fig 1A) and high levels of cholesterol (Fig 1B), although small amounts of cholesterol were also found in fractions 11 and 12. The presence and levels of marker proteins were used to corroborate the isolation of DRM (Fig 1C). Caveolin-1, another protein highly present in DRM involved in Etx oligomerization [39], was also detected. Additionally, western blot analysis of Myelin Basic Protein (MBP) corroborates the presence of myelin in the synaptosomal samples, although in small amounts in DRM fractions (Fig 1C). Thereafter, lipids were extracted from every fraction, as stated in the Materials and Methods section, spotted onto a nitrocellulose membrane and incubated with Etx or proEtx. The bound toxin was detected using a PLO assay, which showed that both Etx and proEtx appear almost exclusively in lipids from the DRM fraction (Fig 1D).

**Fig 2:**
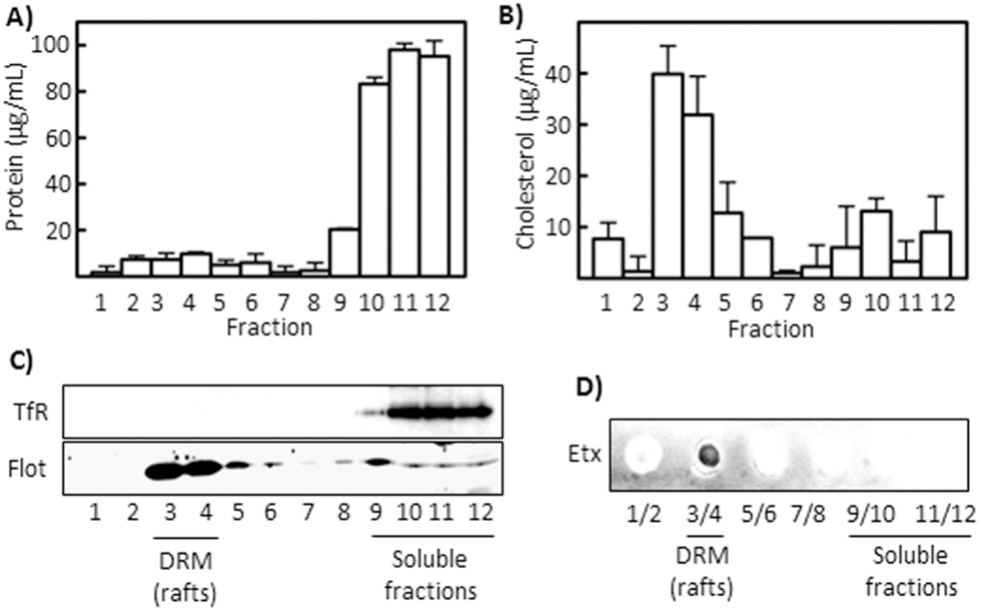
Etx binds to lipids extracted from Triton X-100-DRM (*lipid rafts*) from MDCK cells. Cells were treated with 1% Triton X-100 at 4°C, and detergent-resistant membranes (DRM) were isolated by ultracentrifugation in sucrose gradients. Twelve fractions were harvested after each gradient, and protein (A) and cholesterol (B) contents were determined, showing that the bulk of cholesterol is found in fractions three and four. (C) Marker proteins (Transferrin receptor, TfR, for soluble membranes; Flotillin-1 for rafts) were detected by western blot to prove DRM extraction without contamination from soluble membranes. (D) Lipids, extracted under the Blight and Dyer method, were spotted onto a Hybond-C membrane and overlaid with 2.5 μg/mL of recombinant toxin (PLO assay). Bound toxin was detected with a specific IgG and subsequent chemiluminescence.

The same kind of experiment was performed in MDCK cells, a cell line highly sensitive to Etx and extensively used in the study of Etx activity. The results also show that Etx binds exclusively to lipids present in the DRM fraction from MDCK cells (Fig 2). These results demonstrate that membrane lipids or lipids present in the DRM can act as binding partners of the toxin.

### proEtx binds to phosphatidylserine, mono-phosphoinositides and sulfatide

**Fig 3:**
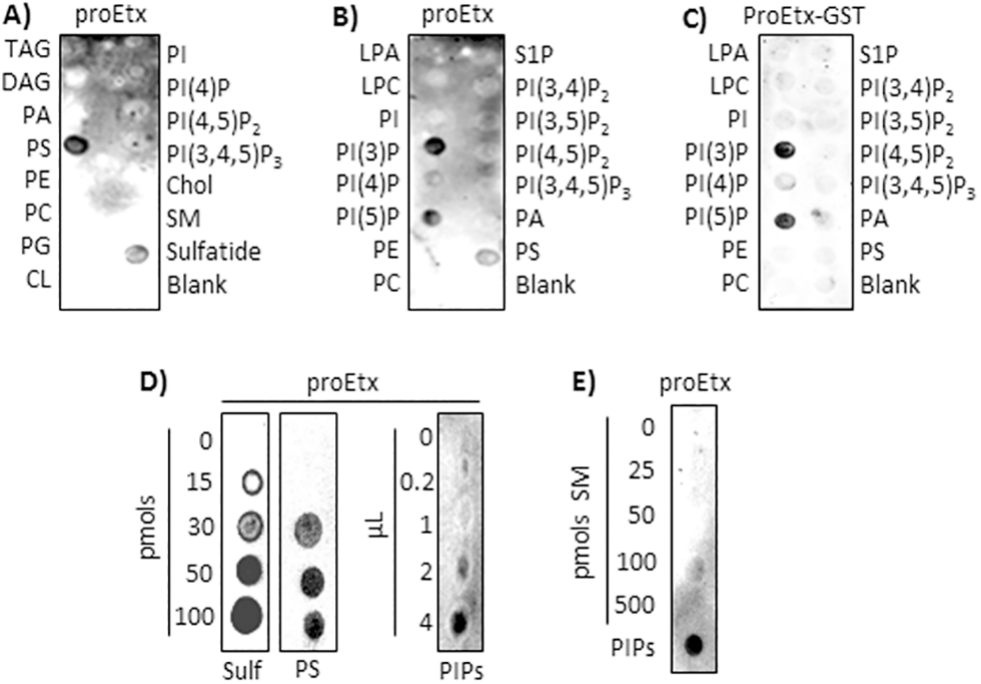
proEtx binds to PS, monophosphorylated PIs and sulfatide. (A) and (B) 2.5 μg/mL of proEtx were overlaid on commercial membranes containing 100 pmols of lipid and detected as in Fig 1. The lipids used were: Triglyceride (TAG), Phosphatidylinositol (PI), PI (4)-phosphate (PI(4)P), PI (4,5)-bisphosphate, (PI(4,5)P2), PI (3,4,5)-trisphosphate (PI(3,4,5)P3), Phosphatidylserine (PS), Phosphatidylethanolamine (PE), Phosphatidic acid (PA), Diacylglycerol (DAG), Cholesterol (Chol), Phosphatidylcholine (PC), Sphingomyelin (SM), Phosphatidylglycerol (PG), 3-sulfogalactosylceramide (Sulfatide), Cardiolipin (CL), Lysophosphatidic acid (LPA), Lysophosphocholine (LPC), and Sphingosine 1-Phosphate (S1P). (C) PLO assays with the same strips as in B were performed with 2.5 μg/mL of proEtx-GST. (D) and (E) Increasing amounts of sulfatide (Sulf), phosphatidylserine (PS), phosphoinositide mixture from bovine brain (PIs) and SM were used to corroborate the presence or absence of proEtx binding. PIs (4 μL) were used as a positive control. Results shown are representative of three independent experiments.

In order to identify the lipids that bind to proEtx, membrane strips spotted with 100 pmols of pure isolated lipids were used in PLO assays with the toxin (Fig 3). The use of PLO assays with membrane lipids showed binding of proEtx to PS and to sulfatide but not to any of the other lipids tested (Fig 3A). The use of membranes with PIs, among others, showed binding of proEtx to phosphatidylinositol-3-monophosphate (PI(3)P), to phosphatidylinositol-5-monophosphate (PI(5)P) and to PS (Fig 3B) only. It is noteworthy that when GST-tagged proEtx was used in the same protocol as in Fig 3B, PI(3)P and PI(5)P were also recognized, but not PS (Fig 3C). In order to corroborate the results shown in Fig 3A and 3B, PLO assays were performed with increasing concentrations of pure sulfatides, pure PS or of a phosphoinositide mix from bovine brain. In every case, the signal due to proEtx binding increased according to the quantity of lipid (Fig 3D). PLO assays with increasing quantities of sphingomyelin were used as a negative control (Fig 3E).

### Presence of mono-phosphoinositides and sulfatide in DRM correlate with their binding to Etx

**Fig 4:**
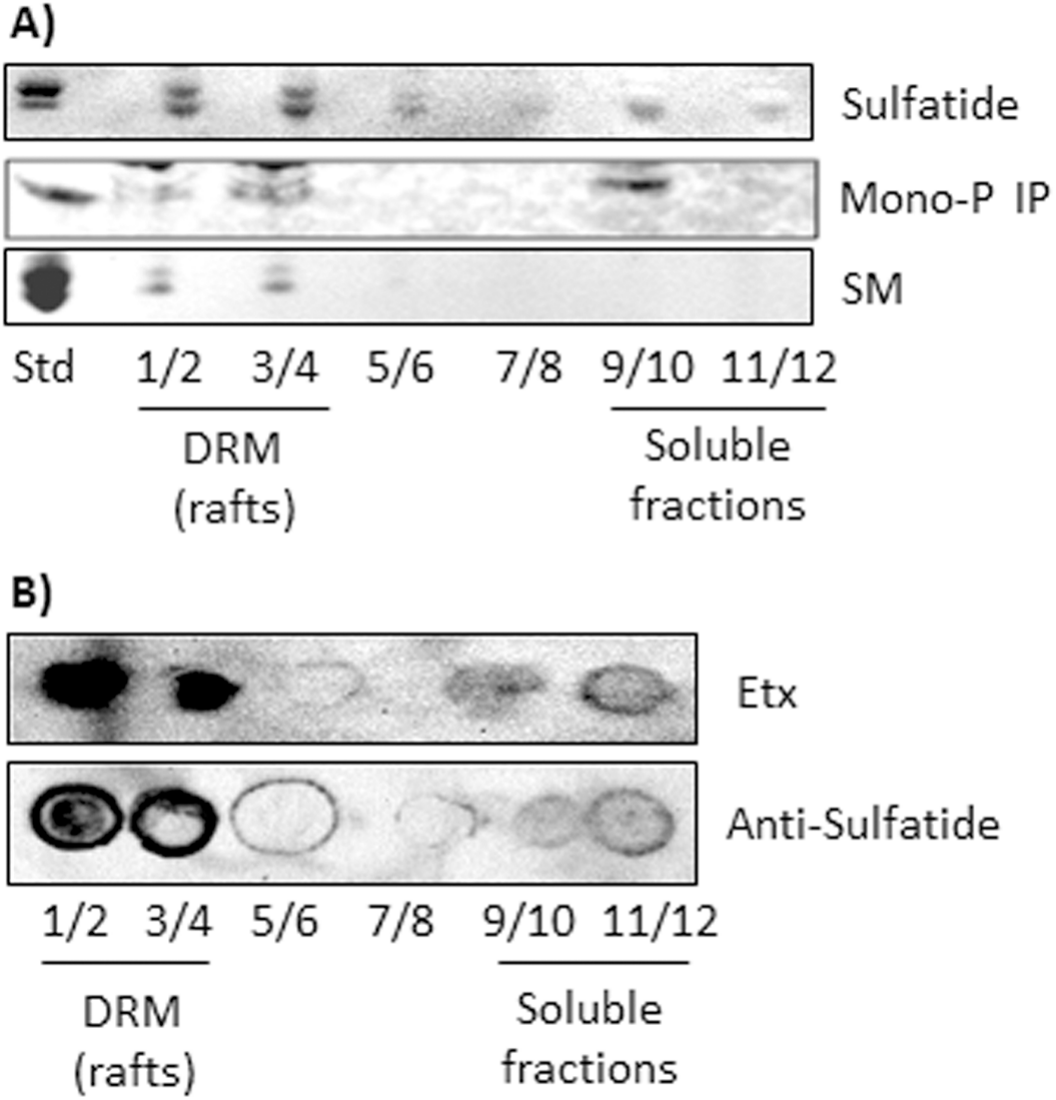
Binding of Etx correlates with the presence of sulfatide and mono-P IPs in DRM. (A) After DRM extraction with ultracentrifugation, the fractions from the gradient were pooled as indicated (1/2, 3/4, etc.), and lipids from the pooled fractions were extracted and resolved with thin layer chromatography, as described in the Materials and Methods section. Standards are shown on the left (Std). SM, sphingomyelin; Mono-P IP, monophosphorylated PI. (B) Lipids, extracted under the Blight and Dyer method, were spotted onto a Hybond-C membrane and overlaid with an anti-sulfatide IgG. The presence of sulfatide in DRM fractions was corroborated and correlates with the binding of Etx to lipids from DRM. Lipids were extracted and processed as in Fig 1D.

Lipids from sucrose gradients from DRM extractions were extracted and separated by TLC, together with standards, in order to study the distribution of some Etx-binding lipids (Fig 4). Sulfatides appeared as a double band and mostly in the DRM fractions. Individual mono-phosphoinositides cannot be separated and identified with TLC, but it was clear that these lipids appeared in two pools, in DRM as well as in the soluble fraction. Sphingomyelin appeared exclusively in DRM fractions (Fig 4A). In the case of sulfatide, its presence in DRM fractions was also analyzed using a monoclonal antibody against sulfatide [51]. The results corroborated the distribution observed in Fig 4A, with sulfatide appearing mostly in DRM fractions (Fig 4B). These results strongly supported the previous results, since the lipid partners of Etx were found in DRM fractions.

### Sulfate group from sulfatides is essential for proEtx binding to lipids from DRM but not for Etx binding to MDCK cells

**Fig 5:**
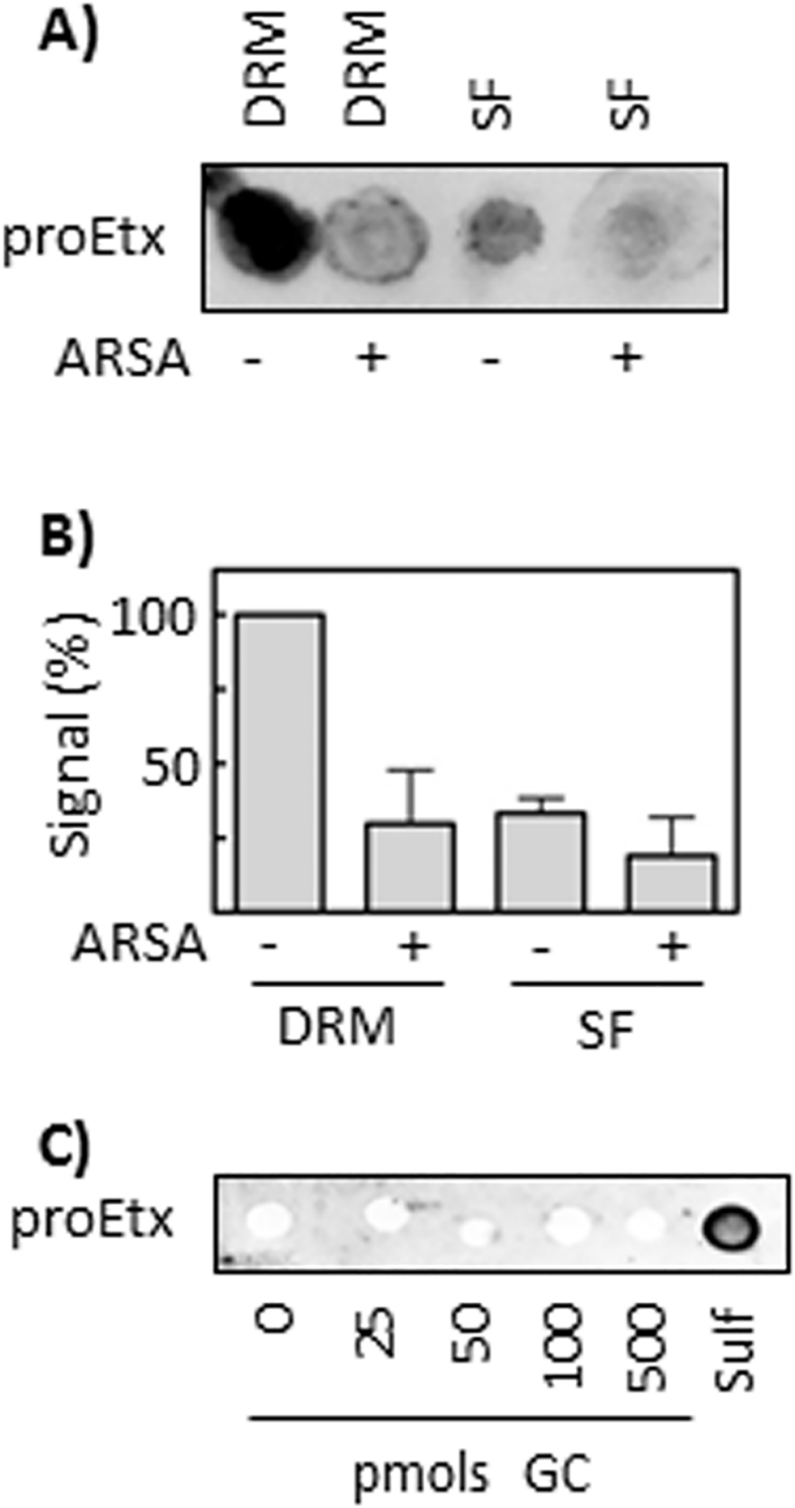
Removal of sulfate group impairs proEtx binding to DRM lipids from synaptosomes. (A) Aliquots from a DRM fraction (#3) and a soluble fraction (#12, SF) from synaptosomal preparations (see Fig 1) were treated with 10 U/mL ARSA, lipids were subsequently extracted and spotted onto a Hybond-c membrane. Binding of proEtx to the processed lipids was then assessed with PLO assay. A representative result is shown. (B) Quantification of the results shown in (A). The signal intensity from untreated DRM samples is taken as 100%. Mean ± SD from three experiments is shown. (C) Binding of proEtx to increasing amounts of galactosylceramide (GC), together with 100 pmols of sulfatide as positive control, was analyzed with the PLO assay. The results show the absence of binding of Etx to GC.

**Fig 6:**
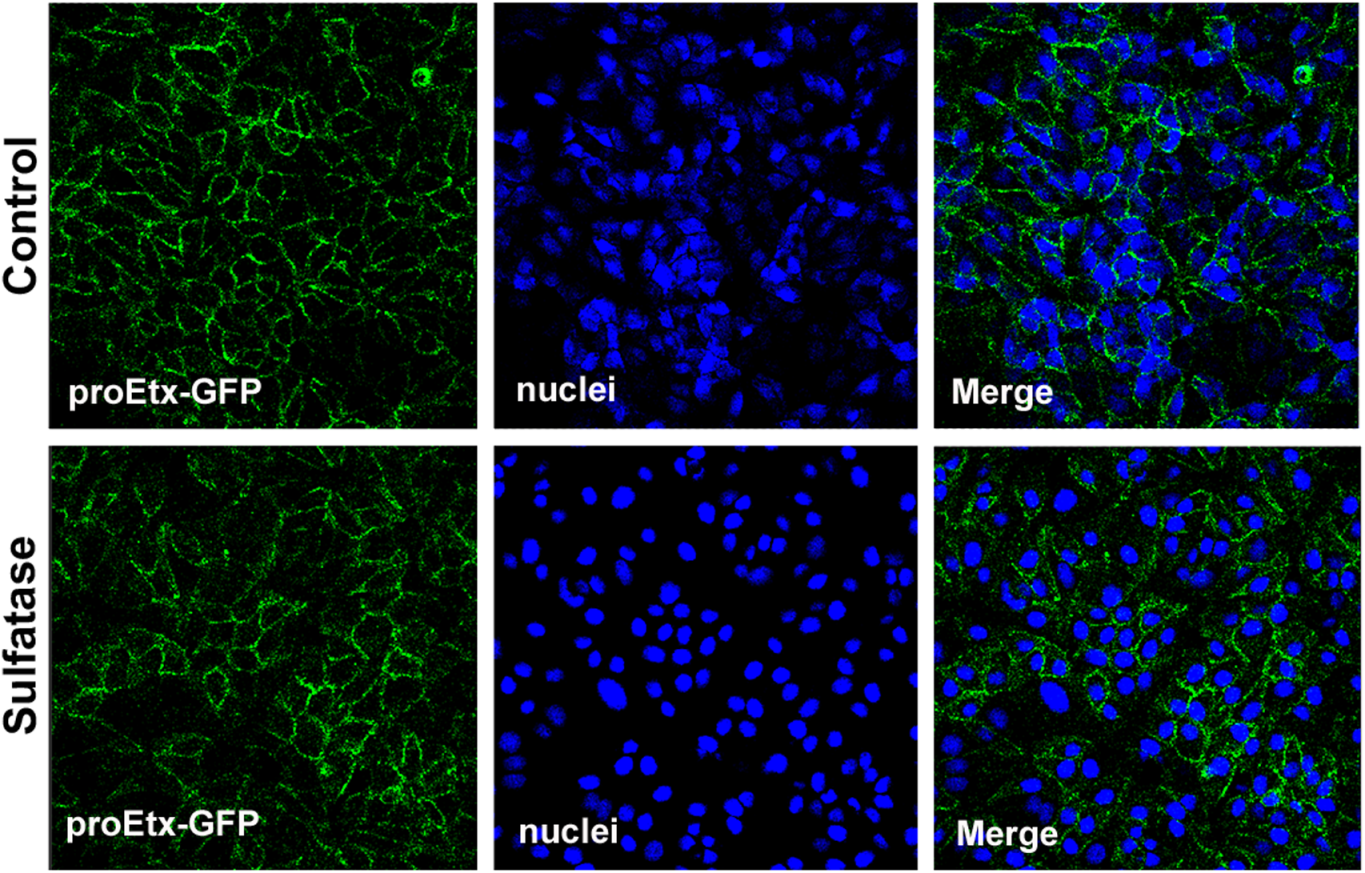
Sulfate group from sulfatides is not essential for proEtx-GFP binding to MDCK cells. Cells were fixed and subsequently treated with 0.25 U sulfatase (1U/mL) for 30 min (at 37°C) or left untreated (Control). Next, both cultures were incubated with 300 nM proEtx-GFP for 1 h at 37°C, and visualized under confocal microscopy. Confocal microscopy images of MDCK cells show the binding of GFP-proEtx to the plasma membrane (green), as well as the nuclei (TOPRO-3 in blue), in the absence or the presence of 0.25 U sulfatase from *Aerobacter aerogenes* pretreatment.

Sulfatase from *Aerobacter aerogenes* and Arylsufatase A (ARSA) are enzymes that specifically cleave the sulfate group present in the sulfatide head of sulfatide. Thus, in order to corroborate results previously shown and to check the role of the sulfate group in proEtx interaction with cellular membranes, aliquots of DRM and of soluble fractions from synaptosomes (fractions 3 and 12 from gradients shown in Fig 1, respectively) were left untreated or treated with ARSA. Subsequently, lipids were extracted using the Blight and Dyer method, and then a PLO assay was performed, using proEtx. The results showed that the removal of the sulfate group from sulfatide strongly impaired proEtx binding to DRM lipids from synaptosomes. Slight binding of proEtx was also observed in the SF fraction, which also decreased under sulfatase pretreatment (Fig 5A and 5B). In order to corroborate these results, binding of proEtx to galactosylceramide (GC), a product of sulfatase enzymatic action on sulfatide, was assayed by means of PLO assay. The results show the absence of binding of proEtx to GC, but a clear binding to sulfatide (Fig 5C). Then, MDCK cultures were treated with sulfatase from *A*. *aerogenes*, prior to incubation with proEtx-GFP. The results showed that the removal of sulfate groups does not significantly abolish proEtx-GFP binding to MDCK cells (Fig 6), indicating that, although proEtx binding to sulfatide can occur in some steps of the toxin–cell interaction, this event is not associated with the primary binding.

### Sulfate removal impairs cytotoxic activity of Etx on MDCK cells

**Fig 7:**
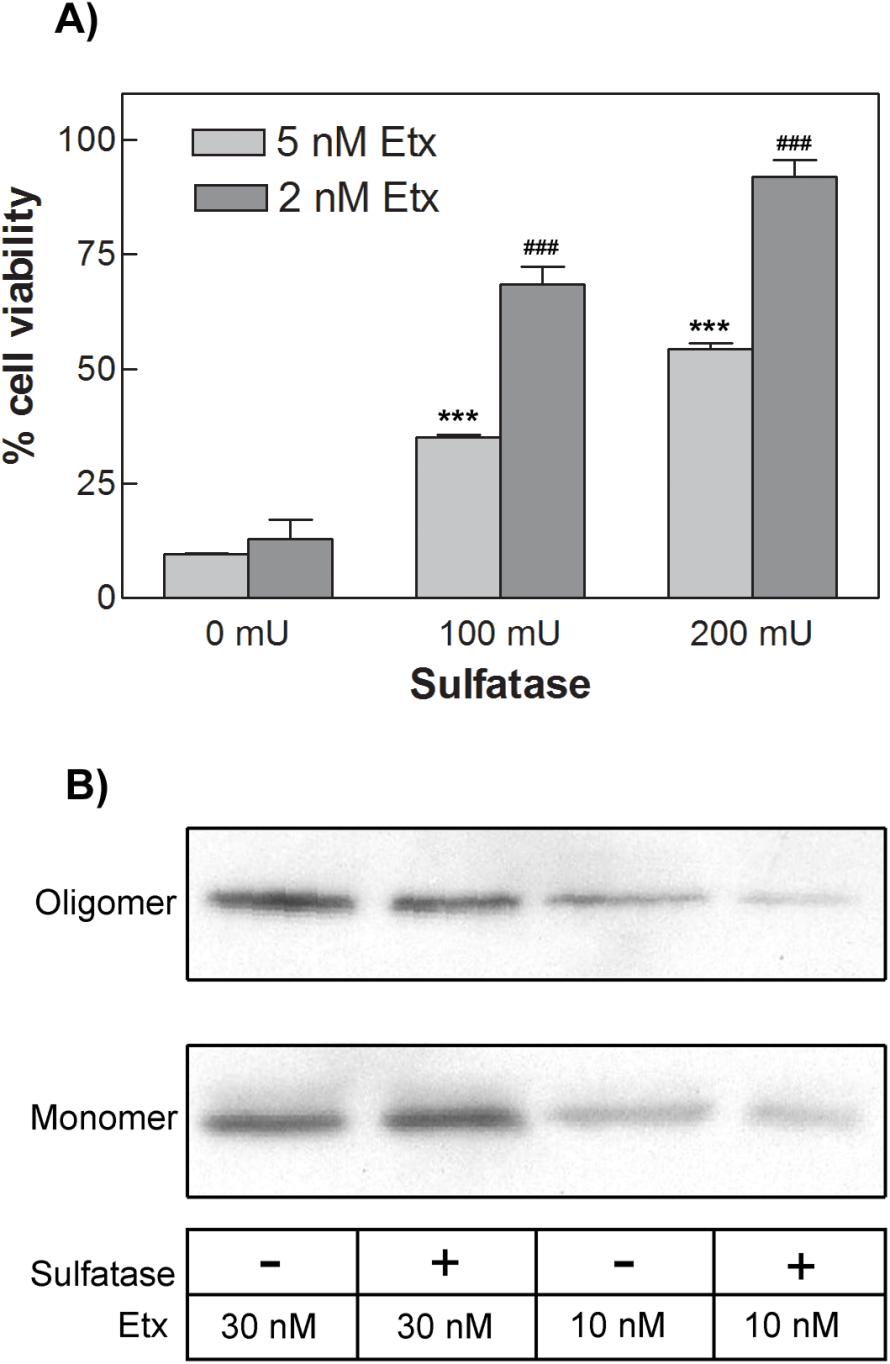
Sulfatase pretreatment of MDCK cells significantly prevents the cytotoxic effect of Etx but does not affect toxin oligomerization. (A) MDCK cells were preincubated in the absence or the presence (0.1 or 0.2 U) of sulfatase from *Aerobacter aerogenes* for 90 minutes at 37°C. Etx was then added at a final concentration of 2 or 5 nM. After 30 minutes at 37°C, MTS assay was used to determine cell viability. Results represent mean ± SEM of cell survival compared to controls (0 U), from three experiments. ***p < 0.001 for 5 nM Etx and ###p < 0.001 for 2 nM Etx, when compared with cells without sulfatase treatment, using one-way ANOVA, followed by the Bonferroni’s post-hoc test. (B) MDCK cells preincubated in absence or presence of 2U/ml of sulfatase were incubated at 37°C with 10 or 30 nM Etx for 30 min. After washing, cells were lysated and the presence of Etx monomers and oligomers analyzed by western blot. The figure shows a representative western blot of three independent experiments.

To define the importance of sulfate groups on the cytotoxic activity of Etx, MDCK cells were incubated with sulfatase from *Aerobacter aerogenes* (100 mU and 200 mU per well of a 96-well plate) before the addition of Etx (2 or 5 nM). The removal of sulfate groups significantly reduced the cytotoxic effect of Etx on MDCK cells (Fig 7), as assessed by MTS assays. This effect was dependent on the concentration of sulfatase and Etx used (Fig 7A). To check if sulfatase pretreatment of MDCK cells would affect the formation of toxin oligomers, MDCK cells were incubated with sulfatase (2 U/ml) for 60 min before Etx addition for further 30 min and the presence of Etx oligomers analyzed by western blot. Although a slight decrease in oligomer formation was apparent after sulfatase treatment (especially in the 10 nM Etx concentration), no significant quantitative differences in Etx oligomeriztion were observed under these experimental conditions compared to the control (no sulfatase treatment) situation (Fig 7B).

## Discussion

Etx is one of the most lethal bacterial toxins, being slightly less lethal than botulinum and tetanus toxin, and produces a rapid cytotoxic effect. Few cell types are susceptible to Etx cytotoxicity, suggesting the presence of a receptor for Etx that is restricted to these cells. Regarding the Etx cellular targets in the nervous system, the binding of Etx-GFP to myelin structures, but not to nerve terminals, from mouse brain has been shown, with identical results for both the non-active prototoxin and the active toxin forms of Etx [11]. Binding to granule cells and to oligodendrocytes from mouse brain has also been shown [14]. The binding of both Etx and proEtx to detergent-insoluble microdomains (DRM, rafts) was described more than ten years ago in MDCK cells and in rat synaptosomes [43]. The results suggested that the toxin is concentrated by binding to an unidentified receptor located preferentially in DRMs and is then induced to form heptameric pores within the rafts [43]. In a later study, the presence of Etx oligomers in MDCK rafts was corroborated, also demonstrating that these Etx-containing rafts are endocytosed and play a crucial role in the Etx-induced vacuolation process [52]. The authors suggested that the primary receptor of monomeric Etx is localized in non-raft regions of cytoplasmic membranes of MDCK cells [52], and that receptors linked with Etx gather in lipid rafts, where Etx forms oligomers. Etx oligomerization is dramatically reduced in cells devoid of caveolins, a group of cholesterol-binding proteins which are enriched in lipid rafts, although the binding of Etx was only slightly perturbed [39], suggesting the presence in the cell membrane of distinct partners of Etx in the binding and oligomerization steps. The results presented here show the virtually exclusive binding of Etx to lipids contained in rafts, with very low binding to non-rafts containing lipids. It is noteworthy that our results on lipid binding were obtained using purified lipids in the absence of membrane structures that could induce toxin oligomerization. The different systems of study might account for variations in the results regarding binding to raft and non-raft domains. One additional possibility would be that rafts containing lipids are directly involved in Etx oligomerization and that the binding of these lipids to Etx would be a subsequent and required step for oligomerization. In any case, we found that both proEtx and Etx bind to the lipid fraction present in DRM membranes from synaptosomal preparations (Fig 1D), suggesting a similar mechanism both in the MDCK and in synaptosomal systems.

One of the Etx-binding lipids identified here is PS, which is mainly found in the inner face of the plasma membrane. However, relatively little is known about binding of proteins from pathogens to PS. *Helicobacter pylori* can cause oncogenic conversion of gastric epithelial cells thanks to the interaction of CagA, a basic protein, with PS in the host cell membrane through a cluster of consecutive lysine and arginine residues, all of which are situated in one domain of the protein [53, 54]. Another case involves the protein EspB from *Mycobacterium tuberculosis*, which can enhance its virulence thanks to its binding to PS [55].

Monophosphorylated inositides are also located in the intracellular leaflet of the plasma membrane. Several intracellular pathogenic bacteria from different species, such as *Listeria*, *Salmonella*, *Mycobacterium* and *Legionella* spp., exploit the phosphoinositide metabolism to infect host cells and to use their signaling, establishing a replicative niche [56, 57]. *Legionella pneumophila* releases a series of proteins that bind to monophosphorylated PIs, such as LidA and LpnE, which bind to host PI(4)P and PI(3)P, interfering with host vesicle trafficking and signaling [58]. Since a series of proteins that bind to PI(3)P, and other PIs, are involved in trafficking of early endosomes, multivesicular bodies and phagosomes [59], some kind of role of the interaction between Etx and monophosphorylated PIs in the vacuolation process described during Etx intoxication can be hypothesized [52]. In any case, the interaction of Etx with monophosphorylated PIs would take place after pore formation, during its internalization process.

Among the lipids herein identified as Etx binding partners, sulfatide is a candidate for mediating the vulnerability of specific host cells to Etx, since sulfatide is abundant in organs targeted by Etx, i.e., kidneys, gastrointestinal tract and the nervous system [60]. In the nervous system, sulfatide accounts for 4% of the total myelin lipids, most of the sulfatide being present in the plasma membrane of oligodendrocytes and Schwann cells, and the myelinating cells of the central and the peripheral nervous systems [61]. The role of sulfatide in the nervous system is not clear, but abnormal levels of synthesis can potentially affect functional properties of membrane proteins, such as transporters, receptors and ion channels, causing neurological symptoms [62]. In myelin structures, sulfatide is essential for paranodal junction formation and for the maintenance of ion channels on myelinated axons [63, 64]. In fact, sulfatide also binds to MAL [65], a protein required for Etx cytotoxic effect on target cells [40]. The binding to sulfatide herein described is consistent with previous work on Etx binding to renal membranes, in which the involvement of O-glycoconjugates, but not of N-glycans, was demonstrated, since β-elimination of O-glycans with alkaline conditions dramatically abrogated proEtx-GFP binding [35]. In fact, sulfatide also binds to many bacteria and to some bacterial proteins [60], as is the case of STb, a heat-stable enterotoxin from *Eschericiha coli* strains [66]. The action of STb results in calcium ion entry in the host cell by means of the opening of a receptor-dependent ligand-gated Ca^2+^ channel [67]. Similarly to STb, Etx induces increased [Ca^2+^]_i_ in both renal cell lines and cultured cerebellar granule cells [14]. These Etx-induced [Ca^2+^]_i_ oscillations are due to the stimulation of mGluR1 metabotropic glutamate receptors, as recently reported in oligodendrocytes [12]. Another relevant case is a protein of the Dps family (DNA-binding protein from starved cells) synthesized by *Campylobacter jejuni* [68]. This Dps protein from *C*. *jejuni* (termed C-Dps) binds to sulfatide on the myelin and the nodes of Ranvier, eventually causing rapid paranodal myelin detachment and down-modulation of sodium channels [68]. These events are thought to be involved in Guillain-Barré Syndrome (GBS) associated with *C*. *jejuni* enteritis [68]. From the structural point of view, C-Dps protein was found to share 41 and 24% amino acid identity with *Helicobacter pylori* neutrophil-activating protein (HP-NAP) and *Escherichia coli* Dps, respectively [69]. GBS, also called polyneuropathy, is caused by demyelination of peripheral nerves, while multiple sclerosis (MS) is caused by demyelination of nerves in the CNS. In any case, since sulfatase treatment does not eliminate proEtx binding nor, apparently, Etx oligomerization in MDCK cultures, but significantly reduces the cytotoxic effect of Etx on these cells, sulfatide binding is likely to be associated with steps leading to cell death, presumably pore formation in raft domains. Sulfatase treatment of MDCK cells prior to Etx incubation substantially decreases Etx induced cell death. However, no significant decrease in oligomer formation was detected with the experimental approach used here. More work is in progress to define the role of sulfate groups in the Etx cytotoxic pathway.

Regarding MS, *C*. *perfringens* type B, an Etx-secreting strain and a non natural host in humans, has recently been isolated from a young individual with first clinical presentation of MS [70]. Since immunoreactivity to Etx in sera and CSF from people with MS is 10 times more prevalent than that from healthy controls, and, moreover, Etx fits mechanistically with nascent MS lesion formation (BBB permeability and oligodendrocyte cell death) the authors postulate that Etx may be a candidate causative toxin for nascent MS lesions. In support of this, the demyelination of organotipic cultures of cerebellar slices triggered by Etx was reported in a recent study [12], without causing the death of oligodendrocytes. Thus, the mentioned work also points to the putative role of Etx in the initial events of certain myelin pathologies. It is noteworthy that this effect is exerted in a manner independent of pore formation and through the activation of an undefined receptor-mediated pathway. Moreover, in a recent work [71] the authors corroborated the Etx-induced demyelination, also in organotypic cerebellar cultures, although Etx caused the selective death of oligodendrocytes in primary cell cultures, which was dependent on MAL expression. Additionally, Etx intoxication results in some MS-like symptoms, such as visual dysfunction, incoordination and spastic paralysis [72]. In fact, the relationship between some sheep-resident pathogens and MS was hypothesized some years ago, since the prevalence of MS is high in global areas where sheep populations are concentrated [73]. The results presented herein add a molecular mechanism, the binding of Etx to sulfatide, which would explain the cellular tropism of action of Etx inside the host and supports the postulated involvement of *C*. *perfringens* type B in the triggering of pathologies caused by demyelination.
